# Differentiated analysis of charismatic leadership personality traits among grassroots managers in universities under the “Double First-Class” initiative

**DOI:** 10.3389/fpsyg.2025.1456093

**Published:** 2025-05-29

**Authors:** Qing-Qing Liang, Lei Liu, Fang Yin

**Affiliations:** ^1^School of Earth Science and Resources, Chang’an University, Xi’an, China; ^2^Shaanxi Key Laboratory of Land Consolidation, School of Land Engineering, Chang’an University, Xi’an, China

**Keywords:** charismatic leadership, personality traits, sensitivity, “Double First-Class” initiative, grassroots managers

## Abstract

This study employs a dual methodology of literature review and questionnaire surveys using the Charismatic Leadership Questionnaire (C-K Scale) to explore the fundamental aspects of charismatic personality traits among 322 grassroots managers across various departments at Chang’an University. The research aims to identify the charismatic personality traits that university grassroots managers should exhibit in the context of the “Double First-Class” initiative. Key findings include: (1) The most significant charismatic leadership trait exhibited by grassroots leaders in universities is their focus on and understanding of their own needs. This is followed by their ability to articulate organizational strategic visions and their sensitivity to internal and external objective environments; (2) Female employees tend to perceive the leader’s charisma more in the dimensions of Strategic Vision Articulation (SVA) and Sensitivity to Member Needs (SMN) compared to male employees. Conversely, male employees are more likely to recognize the leader’s charisma in the Personal Risk (PR) dimension; (3) Male leaders are more inclined than female leaders to demonstrate the personality traits of Personal Risk (PR) and Unconventional Behavior (UB); (4) Leaders aged 30 and below exhibit the most significant behavioral traits in Strategic Vision Articulation (SVA), Self-Efficacy (SE), and Sensitivity to Member Needs (SMN); (5) With an increase in the scope of management, leaders demonstrate more prominent behavioral traits in the Strategic Vision Articulation (SVA) dimension. Leaders overseeing 8–15 people exhibit the most pronounced behavioral traits in the Sensitivity to Member Needs (SMN) dimension. This study contributes to a deeper understanding of the nuanced aspects of charismatic leadership among grassroots managers in academic institutions. It provides insights into how these traits align with the objectives of the “Double First-Class” initiative, offering valuable references for enhancing the leadership capabilities of grassroots managers in universities.

## 1 Introduction

Launched in 2015, China s “Double First-Class” Initiative (双一流建设) aims to cultivate a group of world-class universities and disciplines by 2050, with a focus on enhancing academic excellence, innovation capacity, and global influence (State Council of the People’s Republic of China, 2015). This national strategy places unprecedented demands on university management, particularly at the grassroots level, where departmental leaders play a pivotal role in translating institutional goals into actionable plans. Charismatic leadership, with its emphasis on visionary communication and adaptive behavior(Guo, 2018; Akbarian et al., 2022). Has emerged as a critical competency for navigating the complex challenges of this initiative, such as interdisciplinary collaboration, resource allocation, and talent retention. The pursuit of building a top-tier universities under this initiative-including nurturing exceptional students, fostering interdisciplinary innovation, and contributing global-ready talents—requires leaders to embody not only traditional charismatic traits like self-confidence and vision (Lu and Chen, 2006; Donnellan et al., 2007) but also context-specific competencies such as strategic alignment with national priorities, sensitivity to diverse stakeholder needs, and adaptability to evolving academic landscapes (Cao, 2018; Franssens et al., 2023). However, existing research has rarely examined how these traits manifest among grassroots managers in university departments, whose daily operations are instrumental in advancing the “Double First-Class” objectives (Yun et al., 2015). This study fills this gap by analyzing charismatic personality traits critical for grassroots leaders across university departments, using the C-K Scale to identify how traits like strategic vision articulation and environmental sensitivity align with the initiative’s demand for proactive, goal-oriented management (Lai and Zhao, 2018; Liu et al., 2018a,b).

Against this backdrop, this article explores how charismatic leadership traits-assessed through the Charismatic Leadership Questionnaire (C-K Scale)-enable grassroots managers to drive departmental excellence within the “Double First-Class” framework. By examining gender, age, and team-size differences in trait perception, we aim to provide empirical insights for aligning leadership development programs with the initiative’s goals, ensuring that university leaders possess the competencies needed to navigate its ambitious agenda.

## 2 Literature review

The “Double First-Class” Initiative, since its launch in 2015, has reshaped China’s higher education landscape by prioritizing world-class discipline construction, interdisciplinary innovation, and talent cultivation aligned with national strategic needs (Ministry of Education of the People's Republic of China, 2017). This policy imperative introduces unique leadership challenges for university grassroots managers, who must navigate academic autonomy, resource competition, and stakeholder expectations simultaneously. Charismatic leadership, traditionally studied in corporate or political contexts, takes on distinct significance here: scholars argue that leaders in academic settings must exhibit not only visionary communication (Conger and Kanungo, 1998) but also contextual intelligence to align departmental goals with macro-level policy objectives (Franssens et al., 2023). For example, in disciplines targeted for “first-class” development, managers must balance research excellence with educational equity, a tension that demands traits like adaptive problem-solving and stakeholder sensitivity-qualities often underemphasized in generic charismatic leadership models (Zhao et al., 2020; Wivel and Grøn, 2021).

### 2.1 Leadership theories

Leadership theories have gone through four developmental stages (Table 1):

**Table 1:** Developmental stages of leadership theories (Li, 2017; Wu and Li, 2018).

Stage	Theoretical school	Core concepts
Early 20th Century to Late 1940s	Trait theory of leadership	Focuses on innate personal traits.
Late 1940s to Late 1960s	Behavioral theory of leadership	Emphasizes leaders’ actions and behavioral styles.
Late 1960s to Early 1980s	Contingency theory of leadership	Highlights that different leadership situations require different leaders and styles.
Early 1980s	New leadership theories	Includes ethical leadership, transformational leadership, and charismatic leadership.

### 2.2 Research on personality traits of charismatic leaders

In management studies, ongoing research explores the suitability of individuals for leadership roles. Some researchers argue that certain individuals possess traits that can inspire entrepreneurial spirit, improve company performance, and are recognized as charismatic leaders.

The study of charismatic leadership traits has predominantly taken place in the United States and Canada. Extensive research has demonstrated that leaders’ personality traits significantly impact their leadership styles (Berant et al., 2005; Berghuis et al., 2013; Shang, 2018). A group of scholars, including American leadership theorists such as J. Conger and J. Hay, and Canadian leadership scholar House, have conducted in-depth research on charismatic leadership theory. Their primary objective is to identify the personality traits characteristic of charismatic leaders. They posit that charismatic leaders influence subordinates’ behavior through their personal charisma and further argue that leadership skills can be cultivated and developed through training.

The studies by Conger (1987) and Hay (1986) conducted at McGill University are pivotal in this area. Conger and his colleagues identify key traits of charismatic leaders, including confidence, visionary thinking, the capability to articulate clear objectives, unwavering commitment to those objectives, willingness to engage in unconventional behavior, advocacy for transformative change, and sensitivity to the environment. Hay, on the other hand, proposes that effective leaders should embody nine core traits (Chen and Qu, 2017; Guo, 2018): conceptualization, accountability, adaptability, influence, holistic perspective, foresight, sensitivity to respect, communication skills, and self-awareness.

While charismatic leaders in all sectors are characterized by strong vision articulation (House, 1977), this trait carries unique weight in universities under the “Double First-Class” Initiative. University grassroots managers must translate abstract institutional goals-such as “building global competitiveness” or “fostering interdisciplinary collaboration”-into actionable plans for faculty and staff. A study of Chinese university departments found that leaders who effectively communicated a vision aligned with both academic freedom and policy mandates were more likely to secure stakeholder buy-in, a process that relies on both rhetorical skill and deep disciplinary knowledge (Hu and Shen, 2022). This contextual nuance distinguishes university charismatic leadership from its corporate counterpart, where visions often focus on market dominance rather than scholarly mission fulfillment (Donnellan et al., 2007).

Leadership researchers like Bass (1985) from the United States believe that charisma is a personal quality trait of leaders. Other scholars have found that personality traits such as creativity, adventurousness, confidence, and social sensitivity effectively differentiate charismatic leaders from non-charismatic leaders (House and Howell, 1992). Leaders with proactive personality traits are also more likely to be perceived as charismatic by their superiors. Traditional values, collectivist values, self-transcendence, and self-enhancement values are positively correlated with the formation of charismatic leadership styles. Power motivation is closely related to the formation of charismatic leadership styles (Depoo and Shanmuganathan, 2013). However, scholars like House and others argue that charisma refers to the leader’s ability to profoundly influence the beliefs, values, behaviors, and performance of subordinates through their actions, beliefs, and personality.

Despite ongoing debates, foreign studies generally agree that charismatic leaders can be described by a series of specific personality traits. These traits can typically be identified as follows: Ideal or inspirational goals, the ability to inspire others, behavior that fosters confidence, assertiveness, self-confidence, and a need to influence others. Notably, while prior research has touched on charismatic leadership in higher education (e.g., Bennis and Thomas, 2002), few studies have specifically examined its manifestation among grassroots managers within the “Double First-Class” policy framework. Gaps remain in understanding how demographic factors (e.g., gender, age, team size)-which influence trait perception in general settings (Eagly and Carli, 2007)-interact with university-specific challenges, such as resource scarcity in non-key disciplines or the pressure to publish in high-impact journals. By addressing these gaps, this study aims to refine the C-K Scale for academic contexts and provide evidence-based insights for leadership development programs tailored to China’s higher education reform agenda.

## 3 Research design

### 3.1 Study participants

The survey utilized a peer-evaluation approach, targeting grassroots leaders across various departments at Chang’an University, encompassing teaching, administration, party building, logistics, and other fields. Initially, 400 questionnaires were distributed, with 375 returned, resulting in a response rate of 94%. Subsequently, questionnaires with incomplete responses or signs of careless completion were rigorously filtered out. Specifically, surveys with five or more consecutive identical answers were deemed indicators of non-serious responses and were excluded. After meticulous screening, 53 invalid questionnaires were discarded, leaving 322 valid responses, achieving an effective response rate of 86% (Table 2).

**Table 2:** Basic information of survey participants.

Characteristic variables		Survey participants	
		Number of participants	Proportion(%)
Gender	Male	150	46.6
	Female	172	53.4
Age	30 years old and below	198	61.5
	31–40 years old	98	30.4
	41–50 years old	22	6.8
	51–60 years old	4	1.2
Marital Status	Married	171	53.1
	Unmarried	151	46.9
Education level	High school and below	4	1.2
	Associate’s degree	28	8.7
	Bachelor’s degree	238	73.9
	Master’s degree and above	52	16.1
Years of work experience	Less than 5 years	172	53.4
	5–10 years	100	31.1
	10–20 years	46	14.3
	More than 20 years	4	1.2
Position	Junior Staff	250	77.6
	Team Leader	64	19.9
	Deputy Section Chief / Section Chief	8	2.5
The leader’s level among the management	Senior Management	4	1.2
	Middle Management	4	1.2
	Grassroots / Frontline	314	97.5
Leader’s gender	Male	164	50.9
	Female	158	49.1
Leader’s age	30 years old and below	34	10.6
	31–40 years old	216	67.1
	41–50 years old	64	19.9
	51–60 years old	8	2.5
Scope of leader’s management	1–7 people	78	24.2
	8–15 people	144	44.7
	16–30 people	96	29.8
	31 people and above	4	1.2

### 3.2 Measurement tools

The study adopts a questionnaire survey format structured into two parts. The first part comprises items focusing on demographic variables, while the second part assesses charismatic leadership personality traits using the “C-K Scale” developed by Conger & Kanungo. This scale measures various dimensions of charismatic leadership behaviors, including:

(1) Strategic Vision Articulation (SVA); (2) Sensitivity to the Environment (SE); (3) Sensitivity to Followers’ Needs (SMN); (4) Willingness to Take Personal Risks (PR); (5) Demonstrating Extraordinary Behavior (UB).

The questionnaire has been translated into Chinese for domestic research purposes and has been extensively utilized in numerous studies, demonstrating robust reliability and validity. Respondents rate each item on a 5-point Likert scale.

After adding the scores of each item measuring the personality traits of leaders across dimensions such as Strategic Vision Articulation (SVA), Sensitivity to the Environment (SE), Sensitivity to Followers’ Needs (SMN), Willingness to Take Personal Risks (PR), and Demonstrating Extraordinary Behavior (UB), you can obtain individual scores for each leadership personality trait.

To calculate the total score representing charismatic leadership behavior of the manager, sum up the scores from all dimensions. A higher total score indicates that the measured leader demonstrates more pronounced charismatic leadership behaviors. Conversely, a lower total score suggests that the measured leader exhibits fewer charismatic leadership behaviors.

This approach allows for a comprehensive assessment of charismatic leadership qualities based on the C-K Scale questionnaire, providing insights into the manager’s effectiveness in inspiring and influencing others within the organizational context.

### 3.3 Data analysis

To explore gender differences in demographic characteristics and their potential association with charismatic leadership traits, we conducted cross-tabulation analyses using SPSS 26.0. Chi-square tests were employed to examine statistical significance in the distribution of age, management scope, and position level across genders. For continuous variables (e.g., trait scores), independent samples t-tests were used to compare mean scores between male and female respondents, with subgroup analyses stratified by age or team size where relevant.

As shown in Table 3, female respondents were overrepresented in the 30 years old and below age group (84.9% of female participants vs. 34.7% of male participants), which may partially explain their higher sensitivity to members’ needs, as younger leaders in our sample demonstrated stronger scores in this trait.

**Table 3:** Cronbach’s α coefficient for the survey questionnaire.

Survey scale	Cronbach’s alpha coefficient	Number of items
Overall data	0.908	20
Strategic vision articulation	0.871	6
Sensitivity to the environment	0.782	3
Sensitivity to followers’ needs	0.825	5
Willingness to take personal risks	0.826	3
Demonstrating extraordinary behavior	0.845	3

## 4 Results

### 4.1 Reliability and validity testing

#### 4.1.1 Reliability testing

As illustrated in Table 3, both the overall questionnaire and the five individual factors exhibit reliability above 0.70, with a Cronbach’s *α* coefficient of 0.908 for the total scale. This indicates that the overall questionnaire and the five factors demonstrate strong internal consistency and reliability.

#### 4.1.2 Validity testing

The total variance explained by the five factors is 71.165%, which exceeds 60%. Therefore, it is considered that the validity of the charismatic leadership personality traits questionnaire is good.

Table 4 simultaneously presents the rotated factor matrix, categorizing the 20 item options into five factors, forming the five sub-factors of the scale.

**Table 4:** Orthogonally rotated factor loading matrix.

Feature	Factor 1	Factor 2	Factor 3	Factor 4	Factor 5
1. Proposes inspiring strategies and organizational goals.	0.757	0.255	0.116	0	0.132
2. Inspires by clearly articulating to team members the importance of their work, thereby motivating them.	0.749	0.124	0.145	0.167	0.123
3. Often generates new ideas about the organization’s future.	0.738	0.08	0.21	0.085	0.222
4. Is an inspiring speaker.	0.733	0.065	0.044	0.201	−0.09
5. Demonstrates strategic foresight, frequently proposing various possibilities for the future.	0.721	0.156	0.269	0.01	0.233
6. Has an entrepreneurial spirit and seizes emerging opportunities to achieve goals.	0.72	0.194	0.084	0.324	0.461
7. Is sensitive to opportunities in the environment that contribute to achieving organizational goals (favorable natural and social conditions).	−0.032	0.715	0.282	0.155	0.108
8. Is sensitive to material environmental constraints that may hinder the achievement of organizational goals (such as technological limitations, lack of resources, etc.).	0.208	0.785	0.031	−0.027	0.059
9. Is sensitive to social and cultural environmental constraints within the organization that affect the achievement of organizational goals (such as cultural norms, lack of grassroots support, etc.).	0.1	0.762	0.184	0.173	−0.047
10. Understands the capabilities and skills of other members within the organization.	0.348	0.115	0.746	0.135	0.058
11. Understands the shortcomings and limitations of other members within the organization.	0.127	0.206	0.707	−0.033	0.325
12. Influences others by fostering mutual love and respect.	0.117	0.376	0.525	0	0.152
13. Shows sensitivity to the needs and feelings of other members within the organization.	0.074	−0.036	0.847	0.188	0.235
14. Frequently expresses personal concern for the needs and feelings of other members within the organization.	0.21	0.067	0.822	0.124	0.119
15. Takes high personal risks for the benefit of the organization.	0.18	0.124	−0.179	0.687	0.443
16. Frequently makes high personal sacrifices for the benefit of the organization.	0.361	0.265	0.256	0.684	0.08
17. Takes significant personal risks in pursuing organizational goals.	0.125	0.186	0.458	0.665	−0.156
18. Engages in unconventional behavior to achieve organizational objectives.	0.044	0.343	0.164	0.541	0.593
19. Uses non-traditional methods to achieve organizational goals.	0.373	0.298	0.196	0.147	0.783
20. Frequently exhibits unique behaviors that surprise other members within the organization.	0.012	0.321	0.323	0.259	0.645

Factor 1 categorizes item features 1 to 6 as “Strategic Vision Articulation” (SVA);

Factor 2 categorizes item features 7 to 9 as “Sensitivity to the Environment” (SE);

Factor 3 categorizes item features 10 to 14 as “Sensitivity to Followers’ Needs”;

Factor 4 categorizes item features 15 to 17 as “Willingness to Take Personal Risks” (PR);

Factor 5 categorizes item features 18 to 20 as “Demonstrating Extraordinary Behavior” (UB).

### 4.2 Descriptive statistical analysis of the personality traits questionnaire for charm leadership

Based on Table 5, the descriptive analysis of the Personality Traits Questionnaire for Charm Leadership shows the following results for each factor:

**Table 5:** The descriptive analysis.

Factor	Sample size	Minimum value	Maximum value	Mean (average)	Standard deviation
Formulation and clear articulation of strategic vision	322	1	5	3.42	0.74
Sensitivity to the environment	322	1	5	3.31	0.78
Sensitivity to the needs of subordinates	322	1	5	3.5	0.71
Willingness to take personal risks	322	1	5	2.83	0.91
Exhibit extraordinary behavior	322	1	5	2.66	1.06

Formulation and clear articulation of strategic vision has a mean and standard deviation of 3.42 ± 0.74 points.

Sensitivity to the environment scores 3.31 ± 0.78 points.

Sensitivity to the needs of subordinates scores 3.50 ± 0.71 points.

Willingness to take personal risks scores 2.83 ± 0.91 points.

Exhibition of extraordinary behavior scores 2.66 ± 1.06 points. Among these, sensitivity to the needs of subordinates scores the highest, while exhibition of extraordinary behavior scores the lowest. The ranking of scores for each factor from highest to lowest is as follows: sensitivity to the needs of subordinates > formulation and clear articulation of strategic vision > sensitivity to the environment > willingness to take personal risks > exhibition of extraordinary behavior.

The empirical evidence delineates a hierarchical configuration of charismatic leadership manifestations among academic grassroots leaders. Primary analysis identifies self-actualization orientation-characterized by heightened attention to personal developmental needs-as the predominant behavioral modality. This is succeeded by organizational foresight capacity, encompassing strategic vision formulation and institutional road mapping competencies. Tertiary prominence emerges in environmental perceptiveness, demonstrating acute awareness of both intra-organizational dynamics and sectorial trends. Comparative analysis delineates a relative attenuation in two constituent dimensions: risk propensity in decision-making and demonstration of non-conformist leadership practices, which register lower operational frequency compared to the aforementioned core components.

### 4.3 Comparative analysis of differences in charismatic leadership personality traits

#### 4.3.1 Analysis of gender differences

After conducting a differential analysis based on the gender of the respondents, the T-test results in Table 6 indicate that the *p*-values for the three factors-formulation and clear articulation of strategic vision, sensitivity to subordinates’ needs, and willingness to take personal risks-are all less than 0.05. This suggests that there are significant differences in these three factors between genders. Among these, males scored higher than females on the factor of willingness to take personal risks, while females scored higher than males on the factors of formulation and clear articulation of strategic vision and sensitivity to subordinates’ needs. There were no significant differences observed in the other factors (with corresponding *p*-values greater than 0.05).

**Table 6:** Comparison of charismatic leadership personality traits factors by gender.

Factors	Gender	Sample size	Mean	Standard deviation	*T*-value	*p*-value
Formulation and clear articulation of strategic vision	Male	150	3.382	0.771	−1.02	0.025
	Female	172	3.467	0.721		
Sensitivity to the environment	Male	150	3.24	0.756	−1.704	0.365
	Female	172	3.38	0.797		
Sensitivity to the needs of subordinates	Male	150	3.41	0.761	−2.255	0.014
	Female	172	3.58	0.658		
Willingness to take personal risks	Male	150	2.89	0.985	1.154	0.005
	Female	172	2.77	0.843		
Exhibition of extraordinary behavior	Male	150	2.81	0.986	2.373	0.217
	Female	172	2.53	1.102		

* Indicates *p*-value less than 0.05, ** indicates *p*-value less than 0.01 (similar below).

From this, the following conclusions can be drawn: First, in terms of the formulation and clear articulation of strategic vision and sensitivity to subordinates’ needs, female employees are more likely than male employees to perceive the leader’s charisma. Second, in terms of willingness to take personal risks, male employees are more likely than female employees to perceive the leader’s charisma.

#### 4.3.2 Differential analysis of marital status

After conducting differential analysis on the marital status of the participants, the T-test results from Table 7 indicate: The *p*-values corresponding to the factor of displaying extraordinary behavior are all less than 0.05, indicating significant differences among different marital statuses in this factor. However, no significant differences were observed in the other factors (corresponding p-values all greater than 0.05). Among them, married individuals scored higher than unmarried individuals in the factor of displaying extraordinary behavior toward immediate supervisors. However, due to the project’s p-value being less than 0.05 but with no significant difference in the mean values between the two variables, this paper does not extensively delve into the discussion on this point.

**Table 7:** Comparison of charismatic leadership traits factors across different marital statuses.

Factors	Marital status	Sample size	Mean	Standard deviation	*T*-value	*p*-value
Formulation and clear articulation of strategic vision	Married	172	3.43	0.760	0.069	0.135
	Single	150	3.424	0.729		
Sensitivity to the environment	Married	172	3.27	0.787	−1.068	0.808
	Single	150	3.36	0.773		
Sensitivity to subordinates’ needs	Married	172	3.48	0.686	−0.643	0.622
	Single	150	3.53	0.743		
Willingness to take personal risks	Married	172	2.83	0.916	−0.011	0.743
	Single	150	2.83	0.911		
Display of extraordinary behavior	Married	172	2.71	1.013	0.882	0.002
	Single	150	2.605	1.101		

#### 4.3.3 Analysis of gender differences in leadership

After conducting differential analysis based on leadership gender, the T-test results from Table 8 indicate that both the factors of willingness to take personal risks and displaying extraordinary behavior have *p*-values less than 0.05. This suggests significant differences in these two factors based on leadership gender. Among these, male scores are higher than female scores in both the factors of willingness to take personal risks and displaying extraordinary behavior. No significant differences were observed in the other factors (corresponding *p*-values all greater than 0.05).

**Table 8:** Comparison of charismatic leadership traits among leaders of different genders.

Factors	Marital status	Sample size	Mean	Standard deviation	*T*-value	*p*-value
Formulation and clear articulation of strategic vision	Married	172	3.43	0.760	0.069	0.135
	Single	150	3.424	0.729		
Sensitivity to the environment	Married	172	3.27	0.787	−1.068	0.808
	Single	150	3.36	0.773		
Sensitivity to subordinates’ needs	Married	172	3.48	0.686	−0.643	0.622
	Single	150	3.53	0.743		
Willingness to take personal risks	Married	172	2.83	0.916	−0.011	0.743
	Single	150	2.83	0.911		
Display of extraordinary behavior	Married	172	2.71	1.01	0.882	0.002
	Single	150	2.605	1.103		

Based on this, the following summary can be made:

Firstly, regardless of whether the leader is male or female, employees value the leader’s sensitivity to subordinates’ needs, formulation and clear articulation of strategic vision, and sensitivity to the environment when perceiving their charismatic leadership traits. This point is also corroborated by descriptive statistical analysis of the data. Secondly, male leaders are more likely than female leaders to exhibit behaviors characterized by willingness to take personal risks and displaying extraordinary behavior.

#### 4.3.4 Analysis of educational differences

After conducting differential analysis based on the educational levels of the subjects, the results of the ANOVA test from Table 9 indicate: The *p*-values for the factors of willingness to take personal risks and displaying extraordinary behavior are both less than 0.05, indicating significant differences among different educational levels in these two factors. However, no significant differences were observed in the other factors (corresponding p-values all greater than 0.05).

**Table 9:** Comparison of charismatic leadership traits among different educational levels.

Factors	Sum of squares	df	Mean square	*F*-value	Significance
Formulation and clear articulation of strategic	3.473	3	1.158	2.108	0.099
Sensitivity to the environment	1.978	3	0.659	1.082	0.357
Sensitivity to subordinates’ needs	1.893	3	0.631	1.245	0.293
Willingness to take personal risks	8.081	3	2.694	3.306	0.021
Display of extraordinary behavior	20.554	3	6.851	6.431	0.000

Based on this, the following summary can be made:

Firstly, concerning the factor of willingness to take personal risks, as educational levels increase, employees perceive a lower degree of willingness to take personal risks exhibited by leaders. Secondly, in the factor of displaying extraordinary behavior, employees with college and bachelor’s degrees perceive a higher degree, while those with high school education or below perceive the lowest degree of such behavior exhibited by leaders.

#### 4.3.5 Analysis of differences in years of work experience

After conducting differential analysis based on varying years of work experience among the subjects, the results of the variance test in Table 10 indicate: The establishment and clear articulation of strategic vision, sensitivity to subordinate needs, willingness to take personal risks, and demonstration of exceptional behavior all have *p*-values less than 0.05, indicating significant differences across different years of work experience in these four factors. However, sensitivity to environmental factors did not show significant differences (corresponding *p*-values were all greater than 0.05).

**Table 10:** Comparison of charismatic leadership traits among different years of work experience.

Factors	Sum of squares	df	Mean square	*F*-value	Significance
Formulation and clear articulation of strategic	3.736	3	1.245	2.272	0.08
Sensitivity to the environment	3.566	3	1.189	1.968	0.119
Sensitivity to subordinates’ needs	15.702	3	5.234	11.295	0.000
Willingness to take personal risks	9.819	3	3.273	4.045	0.008
Display of extraordinary behavior	10.956	3	3.652	3.334	0.02

Based on this, the following summary can be deduced. Firstly, in the factors of formulating and clearly articulating strategic vision, and sensitivity to subordinate needs, except for professionals with over 20 years of work experience, the mean values of the other three variables show a gradual increase. However, after reaching a certain number of years of work experience, there is a tendency for these values to decrease instead. Secondly, in the factor of willingness to take personal risks, the mean values of the four variables show a gradual increase. Thirdly, in the factor of demonstrating exceptional behavior, apart from professionals with over 20 years of work experience where the difference is significant, the mean differences among the other three variables are not substantial. This suggests that there is lower recognition and identification of exceptional behavior among leaders with over 20 years of work experience compared to their counterparts with fewer years of experience.

#### 4.3.6 Analysis of differences in leadership age

After conducting differential analysis based on the age differences of evaluated leaders, the results of the variance test in Table 11 indicate: The formulation and clear articulation of strategic vision, sensitivity to the environment, sensitivity to subordinate needs, and willingness to take personal risks all have *p*-values less than 0.05, indicating significant differences across different ages of leaders in these four factors. However, there were no significant differences found in the factor of demonstrating exceptional behavior (corresponding *p*-values were all greater than 0.05).

**Table 11:** Compares charismatic leadership traits across different age groups of leaders.

Factors	Sum of squares	df	Mean square	*F*-value	Significance
Formulation and clear articulation of strategic	6.563	2	3.282	6.103	0.003
Sensitivity to the environment	4.334	2	2.167	3.613	0.028
Sensitivity to subordinates’ needs	6.242	2	3.121	6.349	0.002
Willingness to take personal risks	5.764	2	2.882	3.517	0.031
Display of extraordinary behavior	4.034	2	2.017	1.811	0.165

Based on this, the following summary can be deduced. Firstly, among the factors of formulating and clearly articulating strategic vision, sensitivity to the environment, and sensitivity to subordinate needs, the age group under 30 years old consistently exhibits the highest mean values. Secondly, in the factor of willingness to take personal risks, the mean differences among these four variables are not substantial. However, leaders aged 41–50 show a relatively higher mean value in willingness to take personal risks compared to other age groups.

#### 4.3.7 Analysis of differences in management scope

After conducting differential analysis based on the evaluated leaders’ management scope, the results of the variance test in Table 12 indicate: The formulation and clear articulation of strategic vision, sensitivity to subordinate needs, and demonstration of exceptional behavior all have *p*-values less than 0.05, indicating significant differences among different management levels in these three factors. No significant leadership gender differences were observed in other factors (corresponding *p*-values all greater than 0.05).

**Table 12:** Compares charismatic leadership traits across different management scopes.

Factors	Sum of squares	df	Mean square	*F*-value	Significance
Formulation and clear articulation of strategic	4.577	3	1.526	2.796	0.04
Sensitivity to the environment	4.371	3	1.457	2.422	0.066
Sensitivity to subordinates’ needs	5.327	3	1.776	3.58	0.014
Willingness to take personal risks	0.368	3	0.123	0.146	0.932
Display of extraordinary behavior	11.156	3	3.719	3.396	0.018

Based on this, the following summary can be inferred: firstly, in the factor of formulating and clearly articulating strategic vision, as the number of managers increases, the mean value of this factor also gradually increases. Secondly, in the factor of sensitivity to subordinate needs, the mean value is highest for management scope of 8–15 people. Beyond this range, as the number of subordinates managed increases further, the mean value gradually decreases. Thirdly, in the factor of demonstrating exceptional behavior, the mean value is lowest for management scope of 31 or more people.

## 5 Discussion

Our study, utilizing Conger and Kanungo’s (1998) C-K Scale, uncovers distinct charismatic leadership profiles among grassroots leaders in higher education. As shown in Table 5, ‘sensitivity to followers’ needs’ (*M* = 3.50) emerged as the strongest dimension, surpassing ‘willingness to take personal risks’ (*M* = 2.83) and ‘extraordinary behavior’ (*M* = 2.66). This finding aligns with qualitative research on academic leadership, emphasizing the critical role of trust-building and empathetic communication in navigating collaborative decision-making processes (Kezar, 2014). Notably, male leaders scored higher on risk-taking than female leaders (Table 6), aligning with House’s (1977) classic theory of charismatic leadership, which links assertiveness to leadership perception. However, this contrasts with some gender studies highlighting communal traits in educational contexts (Eagly et al., 1995).

The results indicate that relational and strategic dimensions of charismatic leadership are prioritized in higher education settings, with ‘sensitivity to followers’ needs’ being paramount. This underscores the importance of empathetic communication and trust-building in academic leadership. The higher risk-taking scores among male leaders may reflect broader leadership perception trends but warrant further exploration in the context of higher education’s unique demands. These findings suggest that leadership development programs should emphasize strategic vision articulation and follower sensitivity while acknowledging demographic variations in leadership perceptions. By cultivating leaders who balance institutional ambition with empathetic engagement, universities can strengthen organizational cohesion and drive progress toward academic excellence goals.

## 6 Final conclusion

“In conclusion, this study offers empirical evidence of how charismatic leadership manifests among grassroots university leaders, highlighting the primacy of relational and strategic dimensions over risk-taking or unconventional behaviors. The gender differences in perceived leadership traits (e.g., female leaders’ stronger follower sensitivity, male leaders’ higher risk-taking scores) underscore the need for context-specific leadership frameworks in higher education.

For universities advancing the Double First-Class Initiative, these findings suggest that leadership development programs should prioritize training in strategic vision articulation and follower needs sensitivity, while acknowledging demographic variations in leadership perceptions. By cultivating leaders who balance institutional ambition with empathetic engagement, universities can strengthen organizational cohesion and drive progress toward academic excellence goals.

This research contributes to the limited literature on charismatic leadership in educational settings and provides a foundation for future studies exploring longitudinal impacts of leadership traits on university performance.”

## 7 Management recommendations

### 7.1 Recommendations for leaders

#### 7.1.1 Strengthen strategic vision articulation to drive organizational alignment

To enhance charismatic leadership effectiveness, grassroots leaders should prioritize developing and communicating a compelling strategic vision. Our findings highlight that strategic vision articulation (SVA) is a core dimension of perceived leadership charisma, particularly in aligning team goals with institutional objectives (Table 5). Leaders can:

Cultivate future-oriented thinking by engaging in regular strategic planning sessions, fostering innovative ideas, and articulating long-term goals clearly to subordinates. This not only enhances followership but also cultivates a proactive organizational culture where team members understand their role in advancing institutional missions.

Leverage entrepreneurial mindsets to identify and seize emerging opportunities, such as interdisciplinary collaborations or resource optimization strategies, which demonstrate leadership foresight and inspire confidence in subordinates (Conger and Kanungo, 1998).

#### 7.1.2 Enhance follower sensitivity and environmental awareness for relational leadership

Given that “sensitivity to followers’ needs” (SMN) emerged as the strongest charismatic trait (Table 5), leaders should adopt a people-centric approach to nurture trust and collaboration:

Foster empathetic communication by establishing regular feedback mechanisms (e.g., one-on-one meetings, team workshops) to understand subordinates’ skills, challenges, and aspirations. Proactively addressing their reasonable needs-such as professional development opportunities or work-life balance adjustments-strengthens team cohesion and morale.

Bolster environmental acumen by staying informed about national policies (e.g., the “Double First-Class Initiative”), institutional regulations, and internal cultural dynamics. This sensitivity enables leaders to anticipate operational risks, adapt strategies promptly, and ensure compliance, thereby enhancing their credibility and subordinates’ sense of security.

### 7.2 Recommendations for organizations

#### 7.2.1 Design tailored leadership development programs for diverse employee profiles

Given significant gender and age differences in perceived charismatic traits (Tables 6, 11), organizations should personalize leadership training to address heterogeneous needs:

Integrate demographic insights into curricula, such as emphasizing risk-taking behaviors for female leaders (who scored lower on willingness to take personal risks, Table 6) or enhancing follower sensitivity for male leaders, while recognizing age-related variations in strategic vision preferences (e.g., younger leaders scoring higher on SVA, Table 11).

Develop systematic frameworks that combine theoretical instruction (e.g., charismatic leadership theories) with contextual case studies, ensuring training programs reflect the collaborative and academic nature of university environments.

#### 7.2.2 Align leadership selection with team characteristics and management scope

Given the impact of management scope on leadership traits-e.g., leaders of 8–15 people scoring highest in follower sensitivity (Table 12)-universities should adopt a strategic approach to leadership selection:

Conduct needs assessments to identify the key charismatic dimensions required for different team contexts (e.g., research teams may prioritize innovative behavior, while administrative units may value strategic alignment).

Link selection criteria to institutional goals under the “Double First-Class Initiative,” preferring leaders who demonstrate both visionary thinking (to drive academic excellence) and relational competence (to foster cross-departmental collaboration). This ensures leadership teams are equipped to navigate complex challenges and support long-term organizational objectives.

## Data Availability

The raw data supporting the conclusions of this article will be made available by the authors, without undue reservation.
